# Allelic background of *LEPRE1* mutations that cause recessive forms of osteogenesis imperfecta in different populations

**DOI:** 10.1002/mgg3.21

**Published:** 2013-06-26

**Authors:** Melanie G Pepin, Ulrike Schwarze, Virendra Singh, Marc Romana, Altheia Jones-LeCointe, Peter H Byers

**Affiliations:** 1 Department of Pathology, University of WashingtonSeattle, Washington; 2 Mt. Hope Hospital, University of West Indies Medical SchoolTrinidad and Tobago; 3 Inserm/Université des Antilles et de la Guyane, Centre Hospitalier Universitaire de Pointe-à-PitreGuadeloupe; 4 Department of Medicine (Medical Genetics), University of WashingtonSeattle, Washington

**Keywords:** African, allelic diversity, allelic heterogeneity, *LEPRE1*, mutations, neonatal death, osteogenesis imperfecta

## Abstract

Biallelic mutations in *LEPRE1* result in recessively inherited forms of osteogenesis imperfecta (OI) that are often lethal in the perinatal period. A mutation (c.1080+1G>T, IVS5+1G>T) in African Americans has a carrier frequency of about 1/240. The mutant allele originated in West Africa in tribes of Ghana and Nigeria where the carrier frequencies are 2% and 5%. By examining 200 samples from an African-derived population in Tobago and reviewing hospital neonatal death records, we determined that the carrier frequency of c.1080+1G>T was about one in 200 and did not contribute to the neonatal deaths recorded over a 3-year period of time in Trinidad. In the course of sequence analysis, we found surprisingly high *LEPRE1* allelic diversity in the Tobago DNA samples in which there were 11 alleles distinguished by a single basepair variant in or near exon 5. All the alleles found in the Tobago population that were within the sequence analysis region were found in the African American population in the Exome Variant Project. This diversity appeared to reflect the geographic origin of the original population in Tobago. In 44 individuals with biallelic *LEPRE1* mutations identified by clinical diagnostic testing, we found the sequence alterations occurred on seven of the 11 variant alleles. All but one of the mutations identified resulted in mRNA or protein instability for the majority of the transcripts from the altered allele. These findings suggest that the milder end of the clinical spectrum could be due to as yet unidentified missense mutations in *LEPRE1*.

## Introduction

Osteogenesis imperfecta (OI) is a group of disorders characterized by fractures with minimal or no trauma (Sillence et al. 1979; Rauch and Glorieux 2004). The severity of OI ranges from perinatal lethality to severe skeletal deformities with mobility impairments and very short stature, and to, at the mild end of the spectrum, asymptomatic individuals with a mild predisposition to fractures, normal stature, and normal lifespan. More than 95% of disease-causing mutations for OI have been found in *COL1A1* (MIM# 120150) and *COL1A2* (MIM# 120160), which encode the chains of type I procollagen, the major protein of bone (unpublished data and database of OI mutations [http://www.le.ac.uk/ge/collagen/]). Most of the remainder have biallelic mutations in any of 11 additional genes (Forlino et al. 2011; Byers and Pyott 2012; Rohrbach and Giunta 2012; Pyott et al. 2013) or in *IFITM5* which result in a dominantly inherited form of OI, OI type V (Cho et al. 2012). Mutations in *LEPRE1*, [MIM# 610339], which encodes prolyl 3-hydroxylase 1, account for close to half of individuals with recessively inherited OI (Dalgleish 1997, 1998; see http://www.le.ac.uk/ge/collagen/). On the basis of sequence analysis of a relatively small pool of individuals with perinatal lethal OI, estimate of the causative gene proportions are similar: 95% of infants are heterozygous for a mutation in *COL1A1* or *COL1A2* and the remainder had recessively inherited forms with mutations in other genes (Bodian et al. 2009). One *LEPRE1* mutation (c.1080+1G>T, IVS5+1G>T) has a carrier frequency of about 1/240 in the African American population (Cabral et al. 2009, 2012) and usually results in a perinatal lethal form of OI. As a consequence, about a quarter of African American infants with the perinatal lethal forms of OI are estimated to be homozygous for that single sequence alteration. The c.1080+1G>T allele originated in Ghana and Nigeria in West Africa, where the current carrier frequency ranges from 1% to 2% to as high as 4–5% in individuals from some tribal groups (Cabral et al. 2012). In this study, we sought to determine the carrier frequency of *LEPRE1* c.1080+1G>T in Trinidad and Tobago (T&T). By directed sequencing we discovered extensive allelic diversity in the African-derived Tobago samples studied. This led us to ask if other populations also had a predominant mutant allele and if we could determine the background sequence of normal alleles on which the alterations occurred. We characterized the allelic background on which *LEPRE1* mutations occurred in 44 probands with OI, determined the background sequence on which they occurred, and compared it with a set of normal alleles we identified the 200 individuals from Tobago.

## Materials and Methods

### Consent

Waivers of research consent for human subjects were granted at the University of Washington and University of West Indies, St. Augustine Investigation Review Boards to use anonymized DNA samples from a previous study of newborn blood collected to measure the rate of hemoglobinopathies on Tobago and to review medical records of perinatal deaths.

### Sample collection and sequencing

DNA was extracted from 200 stored newborn screening blood cards in Tobago by standard methods. The samples were collected in 2009 during a pilot test of newborn screening for hereditary hemoglobinopathies. To measure the frequency of the *LEPRE1* c.1080+1G>T allele, a 488-bp fragment of *LEPRE1* that included exon 5 and flanking intron regions was amplified (sense primer: 5′-AAGTAGCAGGCACCAGCTTGT-3′; antisense primer: 5′-TTGAGGCTCCTGTGTACTCCC-3′) and analyzed by automated sequencing (ABI 3130XL). The unique sequence of the amplicons was 445 bp (Chr1:43, 223, 265-43, 223, 710, hg19). The same primers were used for amplification and sequence determination. All variants in the region were recorded and the frequencies were measured.

### Record review

All neonatal death records between 2006 and 2009 at Mt. Hope Hospital in St. Augustine, Trinidad were reviewed by one investigator (M. G. P.). Recorded data included: gestation at birth, cause of death, congenital anomalies, size relative to gestational age, limb length, fractures, radiograph reports, family history, and maternal health history when available.

### Identification of mutations in *LEPRE1* by diagnostic laboratory testing in individuals with OI

In most individuals in whom *LEPRE1* diagnostic gene sequencing was completed in the Collagen Diagnostic Laboratory (CDL), Department of Pathology, University of Washington, the diagnosis of OI was clear on clinical grounds. The strategy of testing was as outlined by van Dijk et al. (2012) so that mutations were first excluded in the type I collagen genes and then mutations were sought in genes known to be associated with recessive forms of OI. The amplification and sequencing primers for *LEPRE1* are available upon request. The sequences of the 445-bp fragment that included exon 5 and the flanking intronic regions from 70 diagnostic samples stored in the CDL were compared to the variants identified in the Tobagonian population. In addition, the homologous sequence of the related gene, *CRTAP* [MIM# 605497], was sequenced and allele frequencies were determined. The frequency of each allele among European Americans and African Americans was identified from the Exome Variant Server (http://evs.gs.washington.edu/EVS/).

## Results

### Neonatal deaths in Trinidad and Tobago

From January 2006 to October 2009, a total of 172 neonatal deaths were documented among an estimated 45,000 births in T&T. With a neonatal death rate of 27 per 1000 births (http://www.who.org) (Bassaw et al. 2001), we would expect to find records of roughly 350 deaths; one third of births occurring at Mt. Hope Hospital. Of 172 neonatal deaths reviewed, none had evidence of neonatal lethal OI.

### Carrier frequency of c.1080+1G>T *LEPRE1* allele in Trinidad and Tobago

Analysis of polymorphic DNA sites in a male population on Tobago determined that 94% are of African descent (Miljkovic-Gacic et al. 2005). In DNA from 200 newborns from Tobago, we identified one sample with a single copy of the *LEPRE1* c.1080+1G>T allele.

### Allelic variants in *LEPRE1*

**Figure 1:**
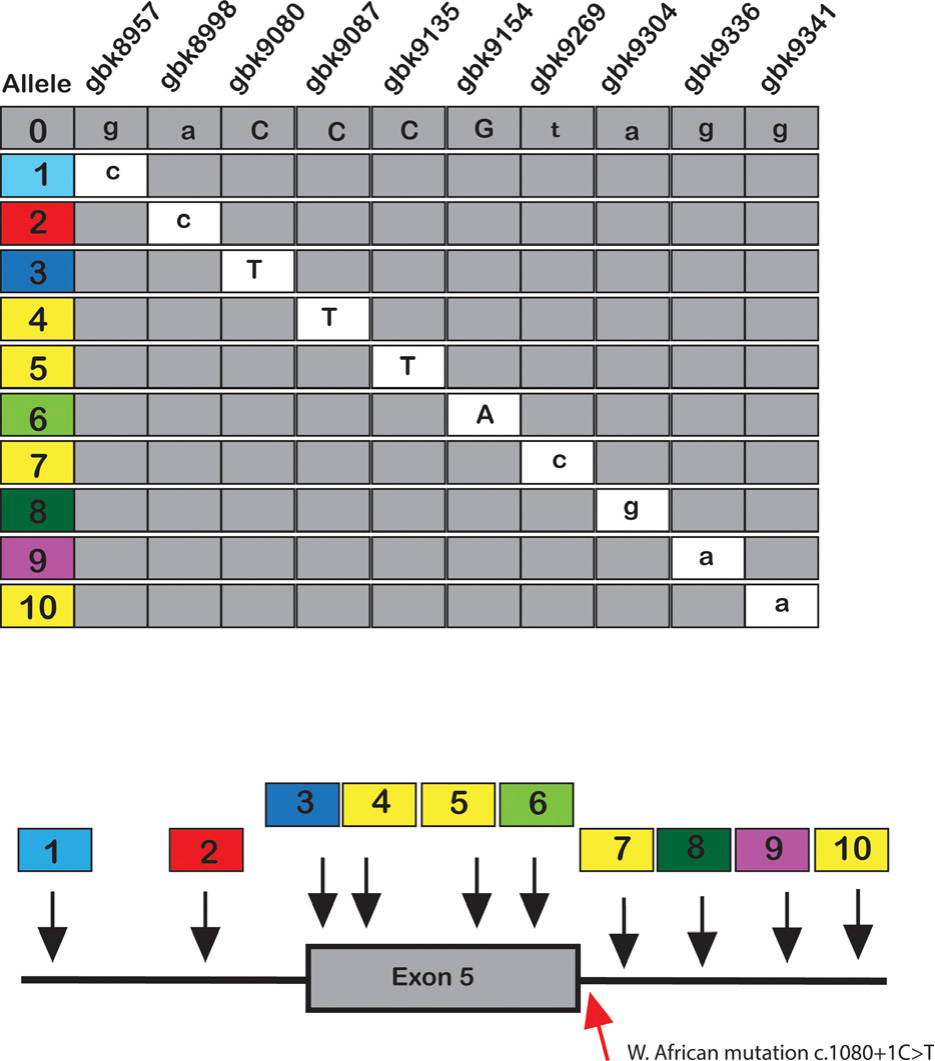
Eleven distinct haplotypes of the amplified fragment were identified in the 200 Tobago samples. No single allele had more than one variant, and each variant occurred on the background of a shared single common allele. Sites 4, 5, 7, and 10 (denoted in yellow) were identified only in Tobago samples.

By sequence analysis of the 445 bp region that encompassed exon 5 and parts of the flanking introns in the 200 Tobagonian newborn samples, we identified 11 *LEPRE1* haplotypes (Fig. 1, Table 1). Each allele was defined by a single basepair variant on the background of a shared sequence. The variants that marked 5 of the alleles were present in the Exome Variant Server (http://evs.gs.washington.edu/EVS/) at about the same frequency in African Americans and in the Tobago population (Table 1). The remaining variants were 80 bp or more from the nearest intron/exon boundary and so were probably beyond the regions sequenced in the EVS sample. Three exonic variants and three intronic variants that we identified in the Tobago population were not present in the clinical diagnostic samples (see Table 1). Five Tobago variants were detected in European and African American populations, and one (c.1045G>A; p.Gly349Arg) was at high enough frequency in both to be consistent with a relatively ancient origin (see Table 1).

**Table 1:** *LEPRE1* gene alleles and frequency in different source samples

Allele	gbk	rs	Chromosome location (Hg19)	Minor allele/major allele	Variant description	Diagnostic samples (140 alleles)	Tobago (400 alleles)	European American	African American
0						111	200		
1	gbk8957	116535864	1: 43223686	c/g	c.941-93G>C	0	c = 15/g = 385	n/a	n/a
2	gbk8998	72956932	1: 43223645	c/a	c.941-52A>C	c = 3/a = 137	c = 65/a = 335	c = 7/a = 4611	c = 336/a = 2318
3	gbk9080	142954359	1: 43223563	T/C	c.971C>T; p.Ala324Val	0	T = 2/C = 398	T = 0/C = 8600	T = 3/C = 4403
4	gbk9087	74070022	1: 43223556	T/C	c.978C>T; p.Thr326Thr	0	T = 22/C = 378	T = 1/C = 8559	T = 179/C = 4227
5	gbk9135	6100157	1: 43223508	T/C	c.1026C>T; p.Ala342Ala	0	T = 14/C = 386	T = 1/C = 8559	T = 191/C = 4215
6	gbk9154	6700677	1: 43223489	A/G	c.1045G>A; p.Gly349Arg	A = 8/G = 132	A = 40/G = 360	A = 374/G = 8226	A = 478/G = 3928
7	gbk9269		1: 43223374	c/t	c.1080+80T>C	0	c = 2/t = 398	n/a	n/a
8	gbk9304	7521929	1: 43223339	g/a	c.1080+115A>G	g = 1/a = 139	g = 24/a = 376	n/a	n/a
9	gbk9336		1: 43223307	a/g	c.1080+147G>A	a = 17/g = 123	a = 14/g = 386	n/a	n/a
10	gbk9341		1: 43223302	a/g	c.1080+152G>A	0	a = 1/g = 399	n/a	n/a

n/a, not identified in the Exome Variant Server (EVS) (http://evs.gs.washington.edu/EVS/).

### Allelic variation in *CRTAP*

We sequenced the same region of the related gene, *CRTAP*, which encodes CRTAP that binds to P3H1, to determine if the allelic variation was similar, given the relationship between the genes. We found only two variants in the Tobago population (c.1032T>G; p.Thr344Thr and c.1044G>A p.Ser348Ser).

### Characterization of and distribution of *LEPRE1* mutations identified in individuals with recessively inherited forms of OI

**Figure 2:**
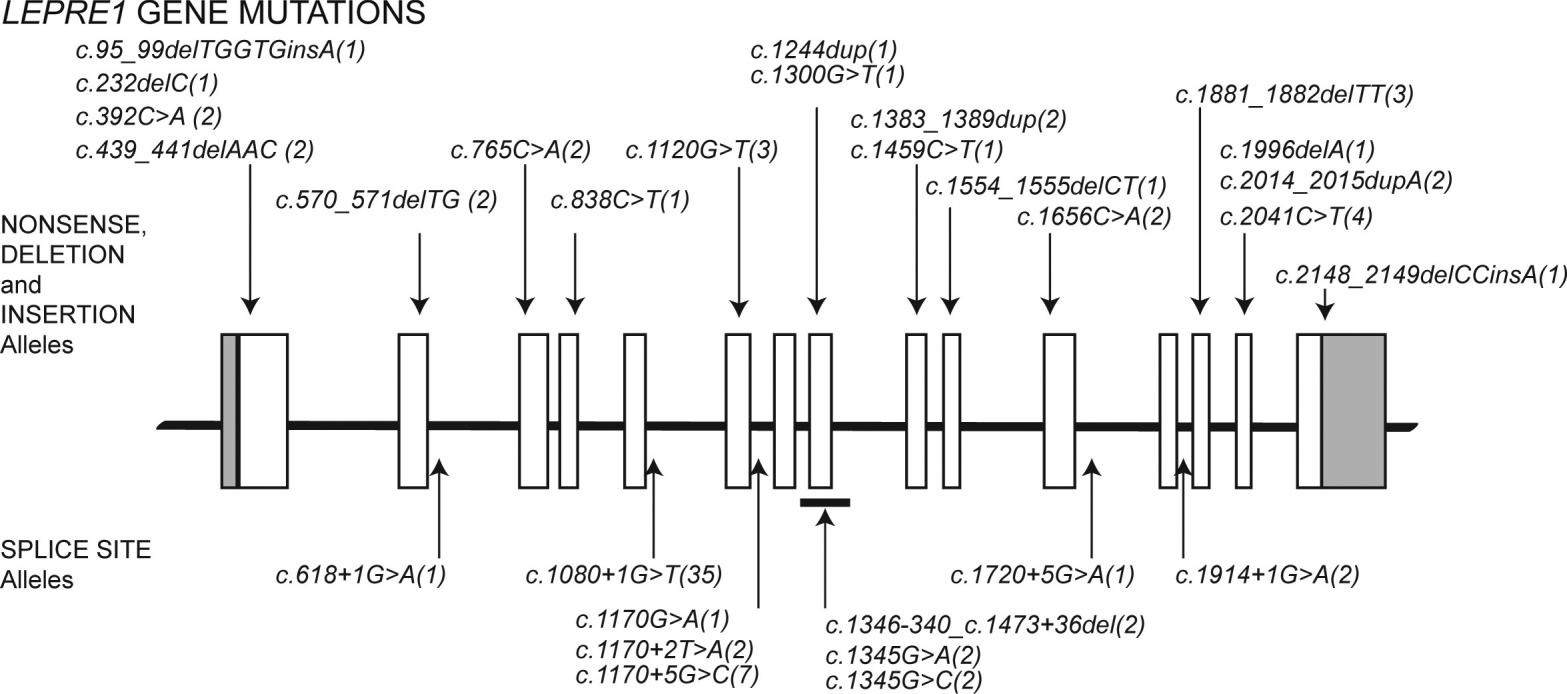
*LEPRE1* mutations identified in Collagen Diagnostic Laboratory University of Washington. Nonsense, deletion, insertion alleles are listed above the gene and splice site mutations and whole exon deletion below. The numbers in parentheses indicate the number of alleles.

Through diagnostic testing, we identified 46 different *LEPRE1* mutations in 44 individuals with recessively inherited OI (Fig. 2, and Tables 2 and 3). Outside of exon 5 and the flanking intronic regions, noncausative sequence variation in the domains we sequenced (exons and flanking intronic regions) was rare. In 29 individuals the identified mutations were homozygous. These included 12 who were homozygous for the c.1080+1G>T mutation, three who were homozygous for the c.1170+5G>C mutation (all of Vietnamese origin), and two who were homozygous for the c.2041C>T mutation who were Arabic, one from Palestine and the other from Saudi Arabia. Each remaining mutation was identified only once.

**Table 2:** Homozygous *LEPRE1* gene mutations

ID	i4-i5 Allele	Allele	Intron or exon	DNA change	Protein	Type	Mutation effect	Ethnicity	Last known age	Previous report
1	9		1	c.392C>A	p.Ser131^*^	Substitution	Nonsense: PTC unstable mRNA – NMMD	Native American	d. I day of age	Proband 7^2^
2^1^	0		1	c.439_441delAAC	p.Asn147del	Deletion	Deletion: deletion of single AA in tetratricopeptide region (stable mRNA product)	Somali	25 years	
3^1^	0		2	c.570_571delTG	p.Gly191Serfs^*^10	Deletion	Frameshift: PTC (exon 2) NMMD	Arabic	3 years	
4	Data needed		5i	c.1080+1G>T		Substitution	Splice Site: results in alternate splice isoforms PTC unstable mRNA NMMD (ref)	African American	1 day of age	
5	2		5i	c.1080+1G>T		Substitution	Splice Site: results in alternate splice isoforms PTC unstable mRNA NMMD	African American	Prenatal ultrasound	Proband 11^2^
6	2		5i	c.1080+1G>T		Substitution	Splice Site: results in alternate splice isoforms PTC unstable mRNA NMMD	African American	Prenatal ultrasound	Proband 12^2^
7	2		5i	c.1080+1G>T		Substitution	Splice Site: results in alternate splice isoforms PTC unstable mRNA NMMD	African American	1 day of age	
8	2		5i	c.1080+1G>T		Substitution	Splice Site: results in alternate splice isoforms PTC unstable mRNA NMMD	African American	Prenatal ultrasound	
9	2		5i	c.1080+1G>T		Substitution	Splice Site: results in alternate splice isoforms PTC unstable mRNA NMMD	African American	3 years	
10	2		5i	c.1080+1G>T		Substitution	Splice Site: results in alternate splice isoforms PTC unstable mRNA NMMD	African American	1 day of age	
11	2		5i	c.1080+1G>T		Substitution	Splice Site: results in alternate splice isoforms PTC unstable mRNA NMMD	African Haitian	2 months	
12	2		5i	c.1080+1G>T		Substitution	Splice Site: results in alternate splice isoforms PTC unstable mRNA NMMD	African American	2 weeks	
13	2		5i	c.1080+1G>T		Substitution	Splice Site: results in alternate splice isoforms PTC unstable mRNA NMMD	African American	Prenatal ultrasound	Proband 14^2^
14	2		5i	c.1080+1G>T		Substitution	Splice Site: results in alternate splice isoforms PTC unstable mRNA NMMD	African American	d. 2 weeks	
15	2		5i	c.1080+1G>T		Substitution	Splice Site: results in alternate splice isoforms PTC unstable mRNA NMMD	African American	12 years	
16	0		6	c.1120G>T	p.Glu374^*^	Substitution	Nonsense: PTC unstable mRNA NMMD	Hispanic	d. 1 day of age	
17^*^	Data needed		6i	c.1170+2T>A	p.Ser361_Pro390del	Substitution	Splice Site: stable product 90 bp deletion	First Nation Canadian	d. 5 years	Proband 18^2^
18	0		6i	c.1170+5G>C	p.Ser361_Pro390del	Substitution	Splice Site: stable product 90 bp deletion	Vietnamese		
19	0		6i	c.1170+5G>C	p.Ser361_Pro390del	Substitution	Splice Site: stable product 90 bp deletion	Vietnamese		
20	Data needed		6i	c.1170+5G>C	p.Ser361_Pro390del	Substitution	Splice Site: stable product 90 bp deletion	Vietnamese	d. 5 months	Proband 19^2^
21^1^	0		8	c.1345G>A	p.Gly449Ser	Substitution	Missense/Splice Site – single AA last nucleotide of exon 8 unstable spliced mRNA products NMMD	Lebanese	2 days of age	
22^1^	6		8	c.1345G>C	p.Gly449Arg	Substitution	Missense/Splice Site – single AA last nucleotide of exon 8 unstable spliced mRNA products NMMD	Finnish	2 months	
23	0		8i	c.1346-340_c.1473+36del	p. (504 bp deletion with breakpoints in introns 8 and 9)	Deletion	Frameshift – PTC in exon 10 unstable mRNA NMMD	French–Canadian	Prenatal ultrasound	Proband 17^2^
24	9		9	c.1383_1389dup	p.Lys464Glufs^*^19	Duplication	Frameshift: PTC in exon 9 unstable mRNA NMMD	Asian Indian	4 years	Proband 16^2^
25	0		11	c.1656C>A	p.Tyr552^*^	Substitution	Nonsense: PTC results in unstable mRNA – NMMD	Pakistani	16 years	Proband 5^3^
26	6		13	c.1881_1882delTT	p.Phe627Leufs^*^4	Deletion	Frameshift: PTC in exon 13 unstable mRNA NMMD	Hispanic	d. 2 weeks	
27^1^	0		14	c.2014_2015insA	p.Ile672Asnfs^*^21	Insertion	Frameshift: PTC exon 15 stable mRNA but without terminus KDEL sequence for anchoring to ER membrane	African American	18 years	
28^1^	0		14	c.2041C>T	p.Arg681^*^	Substitution	Nonsense – PTC P3H1 lacks last 55 AA unstable. (Portion of mRNA probably also unstable)	Palestinian	21 months	
29	Data needed		14	c.2041C>T	p.Arg681^*^	Substitution	Nonsense – PTC P3H1 lacks last 55 AA unstable. (Portion of mRNA probably also unstable)	Arabic	5 months	Proband 9^2^
30	0	Allele 1	1	c.232delC	p.Gln78Serfs^*^30	Deletion	Frameshift: PTC (exon 1) unstable mRNA NMMD	Unknown	Prenatal ultrasound	
	0	Allele 2	13	c.1914+1G>A		Substitution	Splice Site: (disruption of IVS13 donor splice site) outcome unknown			
31	2	Allele 1	5i	c.1080+1G>T		Substitution	Splice Site: alternate splice isoforms PTC unstable mRNA NMMD	African American	Unknown	
	2	Allele 2	1	c.95_99delTGGTGinsA	p.Met32Lysfs^*^24	Deletion/Insertion	Frameshift: PTC (exon 1) unstable mRNA NMMD			
32	2	Allele 1	5i	c.1080+1G>T		Substitution	Splice Site: alternate splice isoforms PTC unstable mRNA NMMD	African American	Prenatal ultrasound	
	0	Allele 2	2	c.618+1G>A		Substitution	Splice Site: (disruption of IVS2 donor splice site)			
33	2	Allele 1	5i	c.1080+1G>T		Substitution	Splice Site: alternate splice isoforms PTC unstable mRNA NMMD	African American	d. 6 weeks	Proband 5^2^
	0	Allele 2	3	c.765C>A	p.Tyr255^*^	Substitution	Nonsense: PTC unstable mRNA NMMD			
34	2	Allele 1	5i	c.1080+1G>T		Substitution	Splice Site: alternate splice isoforms PTC unstable mRNA NMMD	African American	7 months	
	2	Allele 2	3	c.765C>A	p.Tyr255^*^	Substitution	Nonsense: PTC unstable mRNA NMMD			
35	2	Allele 1	5i	c.1080+1G>T		Substitution	Splice Site: alternate splice isoforms PTC unstable mRNA NMMD	Columbian; African American	2 months	Moul et al. (2013)
	0	Allele 2	4	c.838C>T	p.Gln280^*^	Substitution	Nonsense: PTC unstable mRNA NMMD			
36	2	Allele 1	5i	c.1080+1G>T		Substitution	Splice Site: alternate splice isoforms PTC unstable mRNA NMMD	African American	5 months	
	0	Allele 2	6i	c.1170+5G>C	p.Ser361_ Pro390del	Substitution	Splice Site: disrupts IVS 6 donor splice site results in deletion of exon 6 90nts			
37	2	Allele 1	5i	c.1080+1G>T		Substitution	Splice Site: alternate splice isoforms PTC unstable mRNA NMMD	Unknown	1 month	
	0	Allele 2	10	c.1554_1555delCT	p.Phe519Glnfs^*^63	Deletion	Frameshift: PTC unstable mRNA NMMD			
38	2	Allele 1	5i	c.1080+1G>T		Substitution	Splice Site: alternate splice isoforms PTC unstable mRNA NMMD	African American	3 months	
	0	Allele 2	11i	c.1720+5G>A		Substitution	Splice Site: (alternate splice isoforms PTC or exon 11 skip both unstable mRNA NMMD little or no P3H1)			
39	0	Allele 1	5i	c.1080+1G>T		Substitution	Splice Site: alternate splice isoforms PTC unstable mRNA NMMD	Asian; Indian	Prenatal ultrasound	
	0	Allele 2	13	c.1881_1882delTT	p.Phe627Leufs^*^4	Deletion	Deletion: frameshift PTC unstable mRNA NMMD			
40	2	Allele 1	5i	c.1080+1G>T		Substitution	Splice Site: alternate splice isoforms PTC unstable mRNA NMMD	African American	Prenatal ultrasound	
	0	Allele 2	14	c.1996delA	p.Arg666Glyfs^*^29	Deletion	Deletion: frameshift PTC unstable mRNA NMMD			
41	2	Allele 1	5i	c.1080+1G>T		Substitution	Splice Site: alternate splice isoforms PTC unstable mRNA NMMD	African American	1 month	
	0	Allele 2	15	c.2148_2149delCCinsA	p.Glu719Asnfs^*^29	Insertion	Insertion: frameshift PTC unstable mRNA NMMD			
42	0	Allele 1	6	c.1120G>T	p.Glu374^*^	Substitution	Nonsense: PTC unstable mRNA NMMD	European; Italian	Prenatal ultrasound	
	0	Allele 2	8	c.1300G>T	p.Glu434^*^	Substitution	Nonsense: PTC unstable mRNA NMMD			
43	0	Allele 1	6	c.1170G>A	p.Pro390Pro last nucleotide of exon 6 – effect on mRNA splicing	Substitution	Synonymous Splice Site: last nt of exon 6 (greatest effect on IVS6 splicing)	Unknown	3 days of age	
	0	Allele 2	9	c.1459C>T	p.Gln487^*^	Substitution	Nonsense: PTC unstable mRNA NMMD			
44	0	Allele 1	8	c.1244dup	p.Arg416Thrfs^*^40	Duplication	Duplication: results in frameshift PTC unstable mRNA NMMD	Unknown adopted	10 years	
	0	Allele 2	13	c.1914+1G>A		Substitution	Splice Site: predicts disruption of IVS13 donor site (outcome unknown)			

1 Consanguinity.

2 Baldridge et al. (2008).

3 Cabral et al. (2007b).

**Table 3:** Compound heterozygous *LEPRE1* mutations

ID	i4-i5 Allele	Allele	Intron or exon	DNA change	Protein	Type	Mutation effect	Ethnicity	Last known age	Previous report
30	0	Allele 1	1	c.232delC	p.Gln78Serfs^*^30	Deletion	Frameshift: PTC (exon 1) unstable mRNA NMMD	Unknown	Prenatal ultrasound	
	0	Allele 2	13	c.1914+1G>A		Substitution	Splice Site: (disruption of IVS13 donor splice site) outcome unknown			
31	2	Allele 1	5i	c.1080+1G>T		Substitution	Splice Site: alternate splice isoforms PTC unstable mRNA NMMD	African American	Unknown	
	2	Allele 2	1	c.95_99delTGGTGinsA	p.Met32Lysfs^*^24	Deletion/Insertion	Frameshift: PTC (exon 1) unstable mRNA NMMD			
32	2	Allele 1	5i	c.1080+1G>T		Substitution	Splice Site: alternate splice isoforms PTC unstable mRNA NMMD	African American	Prenatal ultrasound	
	0	Allele 2	2	c.618+1G>A		Substitution	Splice Site: (disruption of IVS2 donor splice site)			
33	2	Allele 1	5i	c.1080+1G>T		Substitution	Splice Site: alternate splice isoforms PTC unstable mRNA NMMD	African American	d. 6 weeks	Proband 5^1^
	0	Allele 2	3	c.765C>A	p.Tyr255^*^	Substitution	Nonsense: PTC unstable mRNA NMMD			
34	2	Allele 1	5i	c.1080+1G>T		Substitution	Splice Site: alternate splice isoforms PTC unstable mRNA NMMD	African American	7 months	
	2	Allele 2	3	c.765C>A	p.Tyr255^*^	Substitution	Nonsense: PTC unstable mRNA NMMD			
35	2	Allele 1	5i	c.1080+1G>T		Substitution	Splice Site: splice isoforms PTC unstable mRNA NMMD	Columbian; African American	2 months	Moul et al. (2013)
	0	Allele 2	4	c.838C>T	p.Gln280^*^	Substitution	Nonsense: PTC unstable mRNA NMMD			
36	2	Allele 1	5i	c.1080+1G>T		Substitution	Splice Site: alternate splice isoforms PTC unstable mRNA NMMD	African American	5 months	
	0	Allele 2	6i	c.1170+5G>C	p.Ser361_ Pro390del	Substitution	Splice Site: disrupts IVS 6 donor splice site results in deletion of exon 6 90nts			
37	2	Allele 1	5i	c.1080+1G>T		Substitution	Splice Site: alternate splice isoforms PTC unstable mRNA NMMD	Unknown	1 month	
	0	Allele 2	10	c.1554_1555delCT	p.Phe519Glnfs^*^63	Deletion	Frameshift: PTC unstable mRNA NMMD			
38	2	Allele 1	5i	c.1080+1G>T		Substitution	Splice Site: alternate splice isoforms PTC unstable mRNA NMMD	African American	3 months	
	0	Allele 2	11i	c.1720+5G>A		Substitution	Splice Site: (alternate splice isoforms PTC or exon 11 skip both unstable mRNA NMMD little or no P3H1)			
39	0	Allele 1	5i	c.1080+1G>T		Substitution	Splice Site: alternate splice isoforms PTC unstable mRNA NMMD	Asian; Indian	Prenatal ultrasound	
	0	Allele 2	13	c.1881_1882delTT	p.Phe627Leufs^*^4	Deletion	Deletion: frameshift PTC unstable mRNA NMMD			
40	2	Allele 1	5i	c.1080+1G>T		Substitution	Splice Site: alternate splice isoforms PTC unstable mRNA NMMD	African American	Prenatal ultrasound	
	0	Allele 2	14	c.1996delA	p.Arg666Glyfs^*^29	Deletion	Deletion: frameshift PTC unstable mRNA NMMD			
41	2	Allele 1	5i	c.1080+1G>T		Substitution	Splice Site: alternate splice isoforms PTC unstable mRNA NMMD	African American	1 month	
	0	Allele 2	15	c.2148_2149delCCinsA	p.Glu719Asnfs^*^29	Insertion	Insertion frameshift PTC unstable mRNA NMMD			
42	0	Allele 1	6	c.1120G>T	p.Glu374^*^	Substitution	Nonsense: PTC unstable mRNA NMMD	European; Italian	Prenatal ultrasound	
	0	Allele 2	8	c.1300G>T	p.Glu434^*^	Substitution	Nonsense: PTC unstable mRNA NMMD			
43	0	Allele 1	6	c.1170G>A	p.Pro390Pro last nucleotide of exon 6 - effect on mRNA splicing	Substitution	Synonymous Splice Site: last nt of exon 6 (greatest effect on IVS6 splicing)	Unknown	3 days of age	
	0	Allele 2	9	c.1459C>T	p.Gln487^*^	Substitution	Nonsense PTC unstable mRNA NMMD			
44	0	Allele 1	8	c.1244dup	p.Arg416Thrfs^*^40	Duplication	Duplication: results in frameshift PTC unstable mRNA NMMD	Unknown adopted	10 years	
	0	Allele 2	13	c.1914+1G>A		Substitution	Splice Site: predicts disruption of IVS13 donor site (outcome unknown)			

1 Baldridge et al. (2008).

In the 23 individuals who were homozygous (12) or heterozygous (11) for the c.1080+1G>T mutation all had the sequence alteration on the “2” allele as defined in the Tobago population (Fig. 1, and Tables 2 and 3). In all but one of these the extended haplotype of sampled sequences throughout the gene was identical. In the outlier there was a single nucleotide change (c.941-52c>a) that was consistent either with a second mutation or with a crossover event that transferred the mutation to a “0” allele. Three Vietnamese infants shared the identical homozygous c.1170+5G>C mutation on the background of an identical ancestral “0” haplotype. The Arabic Palestinian mutation was on the background of the “0” allele.

All 44 individuals found by clinical testing to have *LEPRE1*-related OI were identified at birth or by prenatal ultrasound because of the presence of short bowed limbs and multiple fractures. The median age of laboratory diagnosis was 22 days with a range of 1 day to 25 years. The *LEPRE1* mutation was identified later in childhood in about a quarter of the individuals studied although the clinical diagnosis of a moderate to severe form of OI was made in infancy. The delay in testing in those probably represented the restudy of individuals with OI after the recognition of the gene as a candidate for mutations that cause OI. All identified children living with *LEPRE1*-related OI were nonambulatory as a result of bone fragility, bone fracture, and deformity.

In almost all instances, the identified mutations led to premature termination codons and unstable mRNA as a result of nonsense codons, frameshifts, or splice site mutations. The absence of P3H1 that resulted from *LEPRE1* homozygous null mutations was not always associated with a neonatal lethal phenotype (Table 2). Among all the causative mutations that we identified there were only two missense mutations. Each of them changed the same nucleotide (c.1345G>A, p.Gly449Ser and c.1345G>C, p.Gly449Arg), the last nucleotide of exon 8, a position that normally contributes to the function of the splice donor site. In cultured fibroblasts from the individual with the c.1345G>C mutation, we identified four abnormal splice products, three of which contained in-frame premature termination codons (U. Schwarze, unpubl. data) and the fourth was a minor product. The other substitution of the same nucleotide (c.1345G>A) should also affect splicing, but we did not have cells in which to study the outcome. We also identified a synonymous variant (c.1170G>A, p.Pro390Pro) that changed the last nucleotide (G) of exon 6 and is also predicted to affect mRNA splicing. We did not have cells in which to characterize the splice products.

## Discussion

Study of the carrier frequency of the West African *LEPRE1* mutation in Tobago (T&T) established that it is similar to that reported in African Americans, consistent with the origins of the two populations from the same regions of Africa. Sequence analysis of exon 5 and flanking regions in 200 Tobago samples identified 11 alleles that were distinguished by single nucleotide variants in the region. Outside this region, in the sequences we characterized in the course of clinical mutation detection, there was very little diversity. As a result, we could use the variants in the region to identify alleles, follow their migration out of Africa, and determine the background on which each mutation occurred. We identified founder mutations in three additional groups and in each, the mutation was on a distinct allelic background. All the causative mutations we identified, with one exception, led to mRNA instability because of the introduction of a premature termination codon by one of several mechanisms (nonsense mutation, splice site mutations with use of cryptic out of frame splice sites, or frameshift mutations). Identification of an adult with compound heterozygosity that includes the first reported missense mutation suggests that we are likely to continue to find individuals with milder OI phenotypes that will expand the phenotypic range of *LEPRE1*-related OI.

### Recessive inheritance in perinatal lethal OI including *LEPRE1* c.1080+1G>T

Perinatal lethal OI is estimated to have an incidence of approximately 1/40–60,000 births (Sillence et al. 1979). If the population studied by Bodian et al. (2009) is representative recessive forms of OI account for about 5%. This translates into an incidence of 1/800,000 births, consistent with an overall carrier frequency of about 1/450. As the carrier frequency in a population increases, the proportion of infants with recessively inherited OI type II due to homozygosity for one allele increases, as demonstrated in homozygosity for *LEPRE1* c.1080G>T in West Africa and the presence of founder mutations in other distinct geographic endogamous groups.

African ancestry is estimated to be as high as 88% (39,000 of 44,000) on the island of Tobago (http://www.cso.gov/tt/statistics) and about 39% on Trinidad. Slave voyage records (http://slavevoyages.org/tast/database/search.faces) document that approximately 15,000 slaves from West Africa (Bight of Benin, Bight of Biafra and Gulf of Guinea islands [45%], Gold Coast [25%], Sierra Leone [15%], and nearby regions [15%]) disembarked on the island of Tobago. The measured carrier frequency of the *LEPRE1* c.1080+1G>T mutation of one in 200 in Tobago is similar to that seen in the United States among African American individuals which is consistent with the similar African origins of the two populations. Given this carrier frequency, the expected incidence of perinatal OI due to homozygosity for this mutation would be 1 in 160,000 births; one infant with *LEPRE1*-related OI caused by this mutation once every 3–4 years in T&T. The absence of OI neonates in review of the death records is consistent with the rare nature of the disorder, in general, and the identified mutant *LEPRE1* allele frequency in Tobago.

### Allelic diversity identified

The variation seen (11 variants in this 445 bp region of the *LEPRE1* gene) in this population is most consistent with accumulation of sequence alterations among a dispersed population in Africa, incorporation of a diverse population into this small geographic area through the slavery-based migrations, and little or no recombination in the region thereafter. Sequencing of the same region in a related gene (*CRTAP* which encodes CRTAP that interacts with and stabilizes prolyl 3-hydroxylase) found only two variants in the Tobago population leaving us without an explanation for the high sequence diversity in *LEPRE1*.

The *LEPRE1* mutations we identified in diagnostic samples came from individuals of diverse geographic and ethnic backgrounds. Of the mutations we identified, 17 were not previously reported in the database of mutations in OI (http://www.le.ac.uk/genetics/collagen/) (Dalgleish 1997, 1998). Of these mutations, five were seen in the homozygous state and 12 were seen in the presence of a second allele of which 11 were the c.1080+1G>T allele. Given the relative rarity of the mutation bearing alleles, most affected individuals were homozygous for the same mutation and were from discrete ethnic populations. The mutations occurred on the background of four of the 11 alleles that we identified in the Tobago population (alleles 0, 2, 6, and 9). The 1080+1G>T mutation occurred on a single allele [2] with one exception. One infant with parents of SE Indian origin had the mutation on the background of the most prevalent allele [0], consistent with a single recombination event.

### Clinical consequence of biallelic LEPRE1 mutations

Of the 46 different disease alleles we identified, all but four were splice site mutations, nonsense mutations, or led to frameshifts, and all of those were shown or predicted to result in loss of mRNA stability. Two of the remaining mutations likely did not affect mRNA stability but resulted in a protein that lacked the carboxyl-terminal rough ER (RER) localization signal (KDEL sequence). The consequence is functional haploinsufficiency through either rapid protein (P3H1) degradation or lack of P3H1 retention in the RER, or both. One mutation resulted in deletion of a single amino acid. The mRNA was stable, but the fate of the protein remained unclear. Some may have residual function, which could explain the milder phenotype of this individual (ID2, Table 2) relative to all individuals with biallelic *LEPRE1* mutations. The last mutation result in a single amino acid deletion (p.Asn147del). It is striking that of all the reported mutations, only one, true missense mutation (c.1466T>C, p.Leu489Pro) in an individual with a second mutation that disrupted a splice site (Zhang et al. 2012) has been reported. That individual was 24 years old and had a moderately severe form of OI; less severe than those with the other types of mutations in *LEPRE1*. This observation suggests that as the search for recessive OI mutations is widened to include milder phenotypes, this missing class of mutations is likely to appear more frequently.
